# Group Therapy for Patients with Tinnitus at the University of Brasilia Medical School

**DOI:** 10.1590/S1808-86942010000100017

**Published:** 2015-10-17

**Authors:** Lisiane Holdefer, Carlos Augusto C.P. de Oliveira, Alessandra Ramos Venosa

**Affiliations:** 1 Graduate student in Medical Sciences (MSc) - UnB, Speech and Hearing Therapist and Psychologist; 2 Post-Doctorate, Head of Otolaryngology - University Hospital of Brasília; 3 PhD, Professor - University of Brasília, University Hospital of Brasília

**Keywords:** group, behavior therapy, psychotherapy, tinnitus

## Abstract

Although group therapy for tinnitus is a well documented field in the international literature, as far as we know it has never been held in Brazil. This study represents a successful experience of a group therapy for tinnitus based on the tinnitus retraining therapy associated with behavioral cognitive techniques. The goal of the present study is to assess patients with tinnitus before and after the group therapy based on the tinnitus retraining therapy and the behavioral cognitive therapy.

**Materials and Methods:**

Prospective cohort study: 27 subjects signed in for the study, and answered the Tinnitus Handicap Inventory (THI) and the Hospital Depression and Anxiety Scale (HAD), before and after the treatment. We held six structured sessions according to the principles of tinnitus retraining therapy associated with behavioral cognitive techniques.

**Results:**

27 patients started and 19 finished the treatment (8 were taken off). 47.4% men, mean age of 47.6. THI results before and after treatment were respectively: functional: 29 and 14, emotional 24 and 10 and catastrophic 12 and 5 and the HAD scale: anxiety 2 and 9 and depression 10 and 6.

**Conclusion:**

The treatment described is effective in improving tinnitus symptoms.

## INTRODUCTION

Tinnitus is the sound perceived by the person without a sound source in the environment. It is highly prevalent - 15% in the general population and 33% in the elderly^1^. Tinnitus worsens significantly the quality of life of 15% to 25% of the people affected, reducing concentration, sleep, emotional balance and social life^2^.

Jastreboff was the first to describe a neurophysiological model to explain tinnitus, which involves auditory and non-auditory pathways^3^. In this model we have the participation of the limbic system and the autonomous nervous system as determinants of the disorder called tinnitus. The Tinnitus Retraining Therapy - TRT is based on habituation: the brain's capacity to ignore neutral, meaningless stimuli^4^. According to the neurophysiological model, the links between the auditory and the limbic systems are responsible for the emotional reaction triggered by the tinnitus, affecting the autonomous nervous system, causing anxiety, depression and sleep disorders^5^.

TRT is known today as one of the tinnitus treatment with the best results described in the literature. As we set up the multidisciplinary team specialized in tinnitus treatment at a large University hospital in the Otolaryngology Department, treating people with tinnitus, our goal was to apply TRT to all those patients who had not benefited from other treatments for tinnitus. Because of the high number of patients, the TRT individual treatment became impossible. Thus, the combination of Tinnitus Retraining Therapy (TRT) and the Behavioral Cognitive Therapy (BCT) in group therapies became a possibility to be tested^6^.

Tinnitus emotional reactions are considered very important in establishing the discomfort caused by it. Anxiety and depression are frequently described and associated to a greater bother from this sound^7^. The self-administered tinnitus treatment through the Internet, using behavioral-cognitive psychology techniques proved that the intervention by means of the BCT is efficient in the treatment of tinnitus^8^,^9^.

Patients with psychiatric alterations are often times contraindicated for TRT because the instructions are more difficult to be understood and accepted and in these cases, tinnitus treatment is only started after controlling the base disease by the psychiatrist^10^.

Care provided in a public hospital is the treatment of reference for tinnitus in our region, with low financial investment and a large number of patients - who have much to gain from the tinnitus group therapy, since it tends to be useful to meet this demand. This study shows concrete numbers from a pilot successful experience in treating tinnitus in groups combining TRT and BCT.

Although the combined treatment of tinnitus and associated emotional factors is a field of clinical research well documented in the international literature, we could notice that the group treatment for tinnitus with such methodology (TRT and BCT), has not been done in Brazil yet. Thus, we based the treatment proposal hereby discussed on the international literature, adapting the methodology used in these references to the reality of local needs^6^, ^7^, ^8^, ^9^, ^10^, ^11^, ^12^.

This objective study aims at assessing the response to group treatment combining TRT and BCT in tinnitus patients by comparing the questionnaires applied before and after completing the tinnitus treatment program.

## MATERIALS AND METHODS

This study was approved by the Ethics Committee of the School of Health Sciences CEP/FS 015/08. The free and informed consent form was read and signed by all those who took part in this sample.

27 patients started and only 19 finished the treatment, and this last value was the sample used in this study. All the subjects in the study were assessed and selected for the group treatment at the ENT ward of the University Hospital. The indication for such treatment used the following criteria: patients with tinnitus of unknown cause, without success in the prescribed treatment to control the cause; those with symptoms of tinnitus of known etiology however without established treatment, and patients with tinnitus of idiopathic cause. It was not among the selection criteria any prior psychological or psychiatric evaluation, not even of hearing loss, use of hearing aids or current medication. Those who accepted group treatment were included.

The group treatment was based on six structured meetings, lasting for one and a half hour, once a week. The sessions were developed based on TRT associated with BCT. A speech therapist and psychologist coordinated the group treatment. The groups were made up of a maximum of eight (8) patients who came to the weekly sessions for five (5) consecutive weeks and one last session after a two-week interval (total of meetings = 6). Consequently, the THI and HAD were employed once before the group treatment and up to eight (8) weeks of treatment, after the end of the group treatment.

The patients responded the Tinnitus Handicap Inventory - THI13 and the Hospital Anxiety and Depression Scale - HAD). THI is made up of 25 questions, broken down in three scales: functional, emotional and catas-trophical. The functional (F) scale measures the tinnitus interference on mental, social, occupational and physical activities. The emotional scale (E) measures the affective responses such as anxiety, anger and depression. The catastrophic (C) quantifies despair and the incapacity the patient states to deal with the symptom. Three are the response options for each one of the questions, scored as follows: yes (4 points), sometimes (2 points) and not (no points)^14^,^15^. Tinnitus can then be characterized as: negligible (0–16%), mild (18–36%), moderate (38–56%), severe (58–76%) or catastrophic (78–100%)16. the HAD scale is a screening test for depression and anxiety filled out by the patient him/herself. It is made up of 14 questions, seven for anxiety and seven for depression. The patients answered both questionnaires (THI and HAD) before and after treatment.

## RESULTS

The group treatment sessions were setup according to Chart 1.

**Chart 1:** Development of the structured sessions.

	THI and HAD are deployed.
1st session	The group instructions are given. The members are stimulated to talk about their tinnitus and all the important aspects are considered for future needs. Here the patients receive their initial instructions about the principles of the Tinnitus Retraining Therapy (TRT). We then start with sound enrichment.
2^a^ session	TRT session. Slides are used in order to better explain things by means of pictures and diagrams.
3^a^ session	Session based on the tinnitus-related cognitive-behavioral approach.
4^a^ session	Attention control techniques guided to handle tinnitus.
5^a^ session	Relaxation practice (using techniques with the five muscle groups).
6^a^ session	Imagination exercise: associating tinnitus sound to a pleasurable situation. Important aspects are revised and the last doubts are cleared up.THI and HAD deployment.

In all the six sessions the group was stimulated to practice and use, in their own daily routine during the week, what had been discussed in that meeting and the tasks proposed.

The THI and the HAD applied before treatment onset were statistically compared and assessed with the questionnaires filled out after the end of the group sessions.

27 patients started and 19 completed the treatment. 8 subjects were taken out from the sample because they missed treatment sessions. 47.4% were men and 52.6% women. Ages varied between 20 - 78 years (mean of 47.6 years).

THI and HAD results before and after treatment are shown on Table 1.

**Table 1:** THI and HAD results (mean values) before and after the group treatment.

	Pre-treatment (mean)	Post-treatment (mean)	p
Functional THI	29,16	14	< 0.0001
Emotional THI	24,11	10,53	< 0.0001
Catastrophic THI	12,53	5,37	0.0001
Total THI	65,8	29,9	<0.0001
HAD Anxiety	11,84	8,79	0,017
HAD Depression	9,74	6,37	0,0053

## DISCUSSION

Tinnitus can be difficult to treat. New treatment options are always welcome. It is known that among treatment options for tinnitus, TRT is the one with the best results - 82% of improvement after one year^5^.

**Graph 1:**
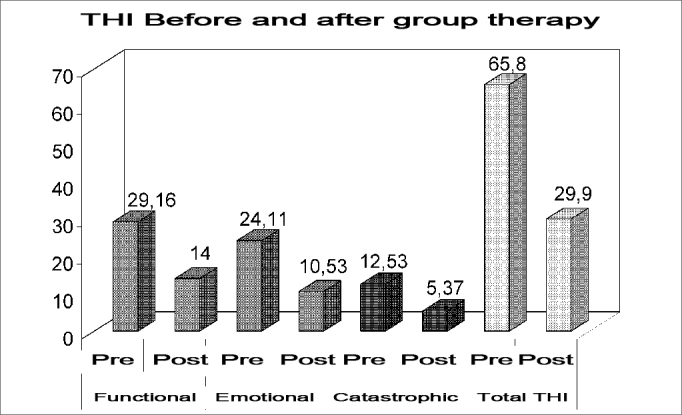
THI mean values (functional, emotional and catastrophic) before and after the group treatment.

**Graph 2:**
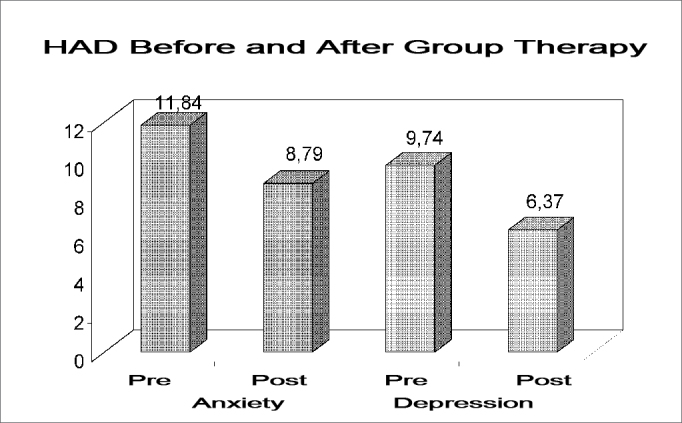
Anxiety and depression mean values before and after treatment in group, assessed according to the HAD scale.

This is a pilot study and the results are encouraging. Despite the small number of patients, the results show an important reduction in the mean scores of tinnitus-related aspects - THI - and the psychological ones (anxiety and depression) - HAD scale - which confirms the efficacy of associating both techniques (TRT and BCT). (Graphs 1 and 2).

The present study suggests that the group treatment for tinnitus, based on TRT and BCT is a good option for tinnitus treatment in public hospitals. Other studies using the same type of techniques also prove the efficacy, regardless of the group selection and the group assessment being different.

Despite the differences in group selection, in the assessment tools and in the number of sessions, all the studies used similar techniques which revealed good results in the application of these tests^1^,^3^.

This study corroborates the findings of the other studies of similar methodologies which used HAD. Londero et al. (2006) assessed the efficacy of the cognitive-behavioral treatment in patients with tinnitus. They used the HAD as an assessment tool which was considered high before treatment (Anxiety (HADa) = 11.6; Depression (HADd) = 7.7). After treatment, the HADa mean value = 7.5 and HADd = 3.46. In the treatment group described in this paper, the HAD mean values before treatment were: HADa = 11.84; HADd = 9.74 and after the group treatment the mean values for HAD were HADa= 8.79 and HADd = 6.377.

The current study suggests that the group treatment for tinnitus in the design used here, joining TRT and BCT is efficient. The results obtained confirm the improvement in tinnitus impact: THI (employed before and eight weeks after the group treatment) showed a significant improvement in functional, emotional and catastrophic tinnitus aspects, as well as the anxiety and depression scores, studied by means of the HAD.

Moreover, it shows good results with improvements in clinical condition in a short period of time: eight weeks only, whilst TRT requires at least 12 months improving tinnitus-related complaints^5^.

Our results match those found in the literature assessing tinnitus treatment through cognitive-behavioral treatment, with methodology similar to the one used in this study^7,^^11,^^12^.

It is a fact that the patients in the present study had financial difficulties and problems as to the days and times of the sessions, which interfered in their coming to the six meetings. This can be the reason why 8 patients missed sessions and broke treatment.

We observed that all the patients who truly followed the instructions and the tasks proposed reported improvement in all the areas of their lives which had been affected by tinnitus before treatment.

## CONCLUSIONS

The results from the tinnitus assessment using HAD and THI show a significant improvement in tinnitus-associated anxiety and depression.

This study suggests that the group treatment with TRT and BCT represents a low cost option for quick and efficient responses in the treatment of tinnitus.
